# Supplementary material for: Chromothripsis during telomere crisis is independent of NHEJ, and consistent with a replicative origin
Source: Genome Res. 2019 May;29(5):737–49. doi: 10.1101/gr.240705.118 (PMC6499312; doi:10.1101/gr.240705.118)
Supplement: Supplemental Material [file supp_gr.240705.118_Supplemental_file_1.zip › contigs/annotated_contigs/DB50/contig.2.DB50_length_803_mean_cov_12.2789539228.docx]

**DB50_length_803_mean_cov_12.2789539228**

```
TGGAGCCTCCTTAGAACTTGCACTGCCGGGCCTCACCCCAGAGTTCAAGAGCCAGCAGCTCCGGGGTGCAGCCAGGAATCTGCATTTCT
 >chr10:1249511-1249905 - E=4e-219 p=6e-03
ATCAACTTCTCAGGTGATGCTCCATGGCTGGCCCAGACATCCCACTTTGAGGTCCACTGAGAAGACAGTCGCTCAGAGCAGCAAGAGAA

CGGTCAAGGGAGGCCCCACTGACCAGCATCACGACCTAACTGGTACCTGACTGTGTTGGCTCCAAATGTGCCGCTTTGCTGCAGAAGTC

TTCAACCCTGGCTCACAGGAGAATCACGTGGGCGGCACCGAGAAAGCTGATGGCCGCACTGACTCCCCAGTGACACCAAAATCCTTGGG

GGAGCCAGGCCTCTGGAGATCCTCAAAGCTCCCAGA|TG|TCCTCTCAGGAGGGTTTGTGCCTCCACTGATCTCCAGGAACTTTATTCC
                                     >chr10:1239128-1239539 + E=2e-235
TAAGAGACCACCGAGGAGTCGGGAGGATGGACGACCGCTCAGGTTCCCCCGAGGTTAAAGTGCAGAGGCTCACAGGAGGCTTCAGAAGA

TGTGAAATTTCCATGGCAGAGGAAAGAATCTGCATTTTTCATTTTTCAGCCTGATAATGACATGACTTTTGGCTTCTGTAAGGATTGTC

ATTATTTCAAAAGATATGGGACAAAGGGACTTTGGGGGAATGGGGTCTTTTGCTGTTCCAAATTATGTATTTTTGTGCCCTAGTACTCA

AAATGTAGTAATATGCCAGGTTTCAAACTGGATAAAAGTGTGGCTGGTTTTCATTGCTCTTCAGTCGGCATTACTCATTTGAGATTGTT

TTTC
```
